# Case Report: Autoimmune polyglandular syndrome type 4 involving diabetes mellitus type 1, autoimmune hepatitis, immune thrombocytopenia, and celiac disease

**DOI:** 10.3389/fendo.2025.1655483

**Published:** 2025-10-15

**Authors:** Miłosz Nesterowicz, Katarzyna Anikiej, Hanna Borysewicz-Sańczyk, Aneta Zasim, Stanisław Trzebuchowski, Katarzyna Bąbol-Pokora, Dariusz Lebensztejn, Elżbieta Leszczyńska, Artur Bossowski

**Affiliations:** ^1^ Department of Pediatrics, Endocrinology, Diabetes with the Cardiology Division, Medical University of Bialystok, Bialystok, Poland; ^2^ Department of Pediatrics, Oncology, Hematology and Diabetology, Medical University of Lodz, Lodz, Poland; ^3^ Department of Pediatrics, Gastroenterology, Hepatology, Nutrition, Allergology and Pulmonology, Medical University of Bialystok, Bialystok, Poland; ^4^ Department of Pediatric Oncology and Hematology, Medical University of Bialystok, Bialystok, Poland

**Keywords:** autoimmune polyglandular syndrome type 4, APS-4, diabetes mellitus type 1, autoimmune hepatitis, celiac disease, immune thrombocytopenia, rare disease, case report

## Abstract

Autoimmune polyglandular syndromes refer to a group of disorders characterized by dysfunction in two or more endocrine glands, often accompanied by autoimmune involvement in non-endocrine tissues. A 6-year-old girl was admitted due to suspected diabetes mellitus. The symptoms observed by the parents were polyuria, polydipsia, and an acetone odor. Physical examination revealed a dry oral mucosa, a tongue coated with a white film, ecchymoses on the upper limbs, and a distended abdomen, arched above the chest level, and increased bowel sounds. After comprehensive differential diagnostics, the patient was identified with diabetes mellitus type 1, autoimmune hepatitis, immune thrombocytopenia, celiac disease, and thus autoimmune polyglandular syndrome type 4. The case illustrates a very rare combination of coexisting autoimmune disorders. When a patient is diagnosed with one autoimmune disease, it is crucial to screen actively for other autoimmune conditions. It is essential to remember that the clinical presentation of a patient is often the result of overlapping symptoms from multiple conditions.

## Introduction

Autoimmune polyglandular syndromes (APS) are a group of autoimmune diseases involving dysfunction of two or more endocrine glands, with a possible autoimmune disorder within another ‘non-endocrine’ organ or tissue. Historically, APS was thought to involve only endocrinopathies, but current understanding recognizes its association with non-endocrine autoimmune disorders (multiple autoimmune syndromes, MAS) (1). Autoimmunopathies, including endocrinopathies, develop under the influence of environmental factors in genetically predisposed individuals. There is a defect in the immune system involving impaired immune tolerance. The immune system erroneously identifies autoantigens, leading to the production of antibodies targeting endocrine organs, typically causing glandular hypofunction (2, 3). The current classification of APS is based on the division developed in 1980 by Neufeld and Blizzard, modified by other authors (4–6). The group includes APS type 1 (APS-1), type 2 (APS-2), type 3 (APS-3), type 4 (APS-4), as well as immune dysregulation, polyendocrinopathy, enteropathy, X-linked (IPEX) syndrome (5, 7). The various components of the syndromes are associated with the presence of specific antibodies in the serum, and the symptoms of each disease may manifest at different times, which may necessitate verification of the diagnosis (5, 6). APS is also sometimes divided into two types: juvenile (APS-1) and adult (including APS-2, APS-3, and APS-4) (1, 8). Meanwhile, immunodysregulation polyendocrinopathy enteropathy X-linked (IPEX) syndrome functions as a separate APS (9). APS-4 involves other combinations of endocrine and non-endocrine autoimmune diseases than those arranged in APS-1, APS-2, and APS-3. It is diagnosed by exclusion, hence. Examples include Addison’s disease + hypogonadism + chronic gastritis or pernicious anemia (Addison-Biermer disease), celiac disease + diabetes mellitus type 1, myasthenia gravis + vitiligo, or diabetes mellitus type 1 + hypogonadism (1, 10, 11).

An important issue in the diagnosis of autoimmunity is to know the location of individual autoantigens in the target organs and their corresponding diseases (Additional file 1). When one organ-specific autoimmune disease is diagnosed, other possible comorbidities should be carefully searched for to prevent the associated potential morbidity and mortality (Additional file 2) (12).

## Case presentation

**Figure 1:**
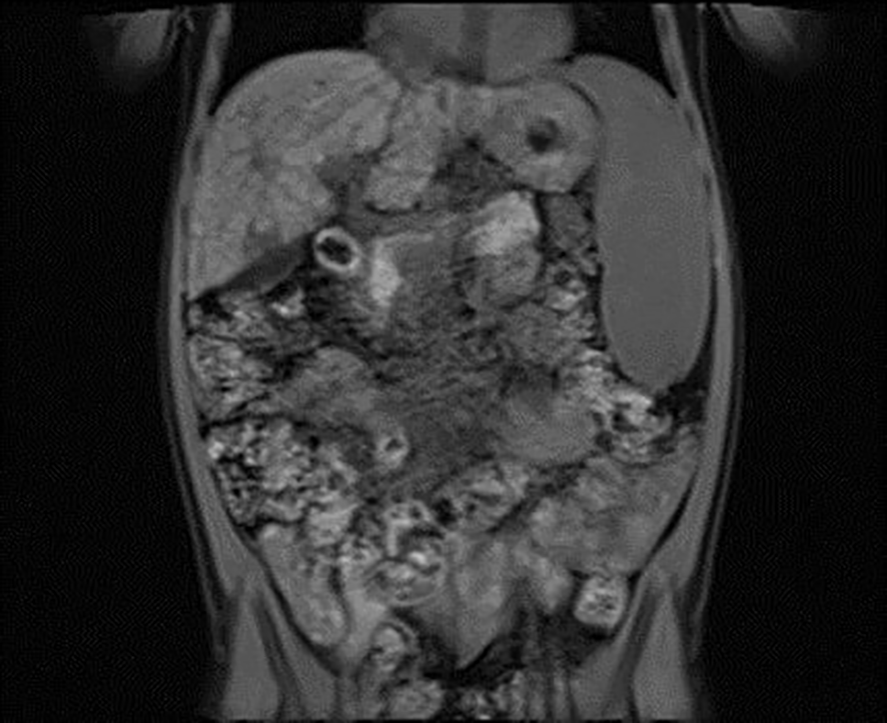
Vertical plane image from magnetic resonance cholangiopancreatography (MRCP) presenting micronodular cirrhosis with moderate biliary changes.

**Figure 2:**
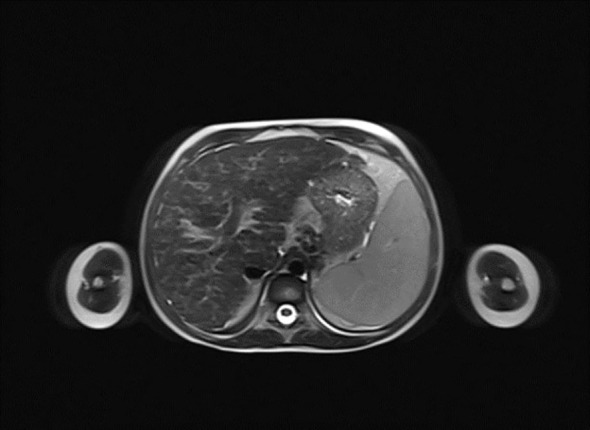
Horizontal plane image from magnetic resonance cholangiopancreatography (MRCP) presenting micronodular cirrhosis with moderate biliary changes.

A 6-year-old girl was admitted to the Department of Pediatrics, Endocrinology, Diabetes with the Cardiology Division due to suspected diabetes mellitus. The parents reported a 4-day history of polyuria and polydipsia, along with a persistent acetone-like breath odor lasting two weeks after a respiratory infection. They did not observe any other concerning symptoms. According to the parents, the patient had no significant prior health issues, was not on any chronic medications, and had no known allergies. The family history was negative for diabetes mellitus. On admission to the Department, the general condition was good, with stable circulatory and respiratory function, logical contact, blood pressure of 91/48 mmHg, and a heart rate of 120 beats per minute (bpm). Physical examination revealed dry oral mucous membranes, a tongue coated with a white film, petechiae on the upper limbs, a distended abdomen, arched above the chest level, and increased bowel sounds. Laboratory findings showed significant abnormalities, including hyperglycemia (578 mg/dL), hyponatremia (128 mmol/L), hypochloremia (93 mmol/L), increased glycated hemoglobin fraction A1c (HbA1c; 9,29%), elevated liver enzyme levels, in gasometry features of compensated metabolic acidosis (pH = 7.38, HCO_3_^−^ = 14.3 mmol/L, base excess [BE] = −9.8). Urinalysis revealed ketonuria (+++) and glucosuria (+++). Peripheral blood counts demonstrated pancytopenia confirmed on two occasions, with white blood cell counts dropping from 3.78 to 1.9 ×10^9^/L, hemoglobin levels from 7.2 to 4 g/dL, and platelet counts from 35,000 to 22,000/μL. Laboratory findings showed coagulation abnormalities, including extended prothrombin time (PT) and activated partial thromboplastin time (APTT), reduced fibrinogen levels, and increased D-dimer concentrations. Diabetes mellitus and diabetic ketoacidosis were diagnosed. The performed additional laboratory tests revealed autoimmune markers for diabetes mellitus type 1, including anti-islet cell antibodies (ICA) at 160 Juvenile Diabetes Foundation (JDF) units and anti-glutamic acid decarboxylase autoantibodies (GADA) at 1779.41 U/mL. Hence, diabetes mellitus type 1 was diagnosed. The treatment included insulin therapy and intravenous electrolyte supplementation, achieving normalization of glycemia and improvement of gasometric values. Subcutaneous insulin therapy and a diabetic diet were initiated on the second day of hospitalization. Abdominal ultrasound revealed mild hepatomegaly and splenomegaly measuring 116 mm. Chest X-ray showed no significant abnormalities. Because of persistent pancytopenia, an oncohematology consultation was conducted. The patient received transfusions of irradiated leukocyte-depleted platelet concentrate and irradiated leukocyte-depleted red blood cell concentrate. Bone marrow biopsy did not indicate a proliferative process but indicated reactive changes. Testing for parvovirus B19 DNA in the bone marrow was negative. Celiac disease was confirmed by highly elevated titers of anti-tissue transglutaminase (tTG) antibodies, anti-deamidated gliadin peptide (DGP) antibodies, and anti-endomysial antibodies (EMA), results exceeded the upper limit of normal by 10-fold. A gluten-free diet was initiated. Microbiological diagnostics, including molecular testing, showed no evidence of active infection. Serological studies demonstrated slightly elevated anti-Epstein-Barr virus (EBV) antibodies in the immunoglobulin (Ig) M (IgM) class and significantly elevated IgG antibodies, along with low anti-severe acute respiratory syndrome coronavirus 2 (SARS-CoV-2) antibody levels. Respiratory antigen testing detected the presence of *Haemophilus influenzae*. The levels of lactate dehydrogenase (LDH) and uric acid were within normal limits. Echocardiography of the heart and thyroid ultrasound showed no abnormalities. Follow-up biochemical tests revealed elevated aminotransferase activity, increased bilirubin levels, decreased albumin levels with normal total protein levels, and slightly elevated ammonia levels. Coagulation assessment showed persistent hypofibrinogenemia, elevated D-dimer levels, and prolonged PT and APTT. Reduced activity of clotting factors VII, IX, XI, XII, and protein C was observed. Following gastroenterological, hematological, and immunological consultations, diagnostics were expanded to include an antinuclear antibody (ANA) panel, which yielded negative results. A significant decrease in complement components 3 (C3) and 4 (C4), elevated levels of IgG (32 g/L) and IgA, normal IgM levels, and normal IgG4 subclass levels were observed, along with lymphopenia in flow cytometry. Subpopulation analysis revealed decreased T lymphocyte counts, including cytotoxic T cells and NK cells, and a reduced percentage of memory T cells. Evaluation of T-cell maturation showed normal percentages of recent thymic emigrants (RTE) and regulatory T cells. B-cell maturation assessment revealed a slightly reduced percentage of switched memory B cells and an increased percentage of IgM-only memory B cells. The percentage of double-negative T cells (DNTC) was slightly elevated. Based on these results, intracellular perforin expression disorders and common humoral and cellular immune deficiencies, as well as complement component deficiencies, were excluded. A panel-based genetic study was ordered to investigate genes whose defects lead to primary immunodeficiency disorders (Additional file 3). Next-generation sequencing (NGS) was performed using the custom-designed SureSelect XT2 Panel (Agilent Technologies, Santa Clara, CA, USA), in a range of genes related to inborn errors of immunity. Sequencing libraries were prepared according to the manufacturer’s protocol and set on the NextSeq 550 platform in the process of a 300-base-pairs (bp) paired-end run (Illumina, San Diego, CA, USA). The data analyses of the target regions were performed using Burrows-Wheeler Aligner Genome Alignment Software and the GATK Variant Caller algorithms and mapped to the human genome reference sequence GRCh37/hg19. The results were next analyzed using Variant Studio v.3.0 (Illumina), SureCall v.4.1 (Agilent Technologies), and Integrative Genomics Viewer v.2.3. The filtering criteria included coverage with at least 20 reads and a minor allele frequency (MAF) below 0.01 in the GnomAD database. Bioinformatic predictions were conducted using the Mutation Taster, SIFT, and PolyPhen platforms. The pathogenicity was determined according to the American College of Medical Genetics (ACMG) classification rules. The analysis did not identify any pathogenic or potentially pathogenic variants. In the anti-tissue antibody panel, positive anti-smooth muscle antibodies (ASMA) and a strongly positive result for anti-liver cytosol antigen type 1 (LC1) antibodies were detected. Differential diagnosis excluded hepatitis A, hepatitis B, hepatitis C, alpha-1 antitrypsin deficiency, and Wilson’s disease, with serum ceruloplasmin levels and urinary copper excretion within normal ranges. IgG4 levels were within normal limits. Urinary bile acid analysis via gas chromatography-mass spectrometry (GC-MS) ruled out inherited metabolic defects. Abdominal Doppler ultrasound excluded portal system thrombosis and revealed a nodular liver with increased stiffness and signs of portal hypertension. Elastography using the S-Shearwave method yielded a METAVIR fibrosis stage F4 – 54.2 kPa, with an interquartile range (IQR) of 18.7%. Gastroscopy excluded esophageal varices and revealed macroscopic changes typical of celiac disease. Magnetic resonance cholangiopancreatography (MRCP) confirmed micronodular cirrhosis with moderate biliary changes (**Figures 1**, **2**). Based on the overall clinical picture and additional test results – significantly elevated alanine transaminase (ALT), hypergammaglobulinemia, high IgG levels, presence of autoantibodies, and exclusion of other causes of chronic liver pathology – autoimmune hepatitis was diagnosed. In the following year, the diagnosis was confirmed by a core needle liver biopsy performed at the Children’s Memorial Health Institute (CMHI). Platelet-bound IgG and IgM on platelets were detected using the direct immunofluorescence (DIF) test. This test, combined with the earlier exclusion of other possible causes of low platelet count, led to the diagnosis of immune thrombocytopenia. Consequently, the patient was diagnosed with a multi-organ autoimmune disease (APS-4; diabetes mellitus type 1, celiac disease, immune thrombocytopenia, autoimmune hepatitis with cirrhosis; **Table 1**).

**Table 1:** Timeline of the diagnostics.

2022/10/31 – 2022/12/13	first hospitalization in the diabetology and endocrinology clinic
2022/10/31	•admission of the patient, a 6-year-old girl, with a 4-day history of polyuria and polydipsia, along with a persistent acetone-like breath odor lasting two weeks after a respiratory infection •physical examination: dry oral mucous membranes, a tongue coated with a white film, petechiae on the upper limbs, a distended abdomen, arched above the chest level, and increased bowel sounds •laboratory findings: hyperglycemia (578 mg/dL), hyponatremia (128 mmol/L), hypochloremia (93 mmol/L), elevated liver enzyme levels, features of compensated metabolic acidosis (pH = 7.38, HCO_3_^−^ = 14.3 mmol/L, base excess = −9.8), ketonuria (+++), glucosuria (+++), pancytopenia (decreased white blood cell count [3.78 ×10^9^/L], hemoglobin level [7.2 g/dL], and platelet count [35,000/μL]), extended prothrombin time and activated partial thromboplastin time, reduced fibrinogen levels, and increased D-dimer concentrations, lactate dehydrogenase and uric acid within normal limits •diabetes mellitus and diabetic ketoacidosis diagnosis •abdominal ultrasound: mild hepatomegaly and splenomegaly
2022/11/1	•chest X-ray: lack of any significant abnormalities •laboratory findings: anti-islet cell antibodies at 160 Juvenile Diabetes Foundation units, anti-glutamic acid decarboxylase autoantibodies at 1779.41 U/mL, 10-fold exceeding the upper limit of normal titers of anti-tissue transglutaminase antibodies, anti-deamidated gliadin peptide antibodies, and anti-endomysial antibodies •diabetes mellitus type 1 and celiac disease diagnosis
2022/11/2	laboratory findings: slightly elevated anti-Epstein-Barr virus antibodies in the immunoglobulin (Ig) M (IgM) class and significantly elevated IgG antibodies, low anti-severe acute respiratory syndrome coronavirus 2 antibody levels; presence of *Haemophilus influenzae* in respiratory antigen testing
2022/11/4	laboratory findings: significant decrease in complement components 3 and 4, elevated levels of IgG (32 g/L) and IgA, normal IgM and IgG4 subclass levels, lymphopenia in flow cytometry (decreased T lymphocyte counts, including cytotoxic T cells and NK cells, reduced percentage of memory T cells, normal percentages of recent thymic emigrants and regulatory T cells, slightly reduced percentage of switched memory B cells, increased percentage of IgM-only memory B cells, slightly elevated percentage of double-negative T cells
2022/11/8	bone marrow biopsy: lack of proliferative process, reactive changes, negative testing for parvovirus B19 DNA
2022/11/9	antinuclear antibody panel: negative results
2022/11/10	abdominal Doppler ultrasound with S-Shearwave elastography: portal system thrombosis exclusion, nodular liver with increased stiffness and signs of portal hypertension indication, METAVIR fibrosis stage F4 (54.2 kPa) with an interquartile range of 18.7% determination
2022/11/14	•laboratory findings: positive anti-smooth muscle antibodies and a strongly positive anti-liver cytosol antigen type 1 antibodies in the anti-tissue antibody panel, significantly elevated alanine transaminase, hypergammaglobulinemia, high IgG levels, the presence of autoantibodies •other causes of chronic liver pathology exclusion •autoimmune hepatitis diagnosis
2022/11/16	gastroscopy: esophageal varices exclusion and macroscopic changes typical of celiac disease indication
2022/11/22	panel-based genetic study of defects leading to primary immunodeficiency disorders: lack of identification of any pathogenic or potentially pathogenic variants
2022/12/6	magnetic resonance cholangiopancreatography: micronodular cirrhosis with moderate biliary changes confirmation
2023/4/5	•direct immunofluorescence test: platelet-bound IgG and IgM on platelets detected •autoimmune pancytopenia diagnosis
2023/4/11	core needle liver biopsy: autoimmune hepatitis confirmation

Ig, immunoglobulin.

Treatment included intravenous antibiotic therapy (amoxicillin with clavulanic acid), parenteral hydration, albumin infusions, vitamin K, folic acid, and vascular endothelium-sealing agents. Due to reported abdominal pain, drotaverine hydrochloride and simethicone were administered as needed. In light of persistent pancytopenia in peripheral blood morphology, coagulation disorders, elevated liver enzyme activity, and lack of effect from immunoglobulin administration (at a dose of 0.4 g/kg body weight over 5 days), treatment with prednisone (2 mg/kg body weight) was initiated, divided into two doses of 20 mg each per day. Given the planned long-term steroid therapy, a densitometry test was performed to assess baseline bone mineral density. During hospitalization, the child’s condition remained stable, with weight gain, improved bowel movement regularity, reduced abdominal bloating, and regression of bruising at insulin injection and blood draw sites. Following the initiation of steroid therapy, significant hyperglycemia requiring substantial increases in insulin doses was observed. Consequently, a personal insulin pump was connected, improving glycemic profiles and reducing insulin requirements. The child’s mother was trained in intensive insulin therapy, the nature of the disease, a diabetic diet with carbohydrate exchange units, dose verification, and management in specific situations. She was also technically trained in insulin pump operation, including basal rate modification, insulin-to-carbohydrate ratio adjustments, bolus calculator use, and handling special circumstances (e.g., physical activity, hypoglycemia). After completing examinations, normalizing glycemia, and gradual improvement in laboratory parameters, the patient was discharged in good general condition with a recommendation for continued treatment in outpatient settings in diabetology, endocrinology, gastroenterology, hematology, and immunology clinics (**Table 1**).

## Perspective of the patient’s parents

‘Before her diagnosis, our daughter was a very active and cheerful girl. Over time, however, she began to experience serious intestinal problems, and the disease and intensive steroid treatment caused severe weakness, apathy, and loss of self-confidence. She tired quickly, had no desire to play or be active, and became withdrawn. Long hospitalization and the need for treatment meant that she had to receive individual tuition, and subsequent frequent illnesses and hospital visits caused her to fall behind in her education and repeat the third grade.

The treatment that was implemented, and in particular the use of an insulin pump with a loop, brought about a huge improvement. Our daughter regained her well-being, zest for life, and energy. She now goes to school, is doing better and better, practices acrobatics, and is once again a lively, active girl. The beginnings of a gluten-free diet were very difficult, but over time, we learned how to follow it, and now we try to enjoy life to the fullest, making sure to maintain our daughter’s good health and well-being’.

## Discussion

APS-4 is a rare condition characterized by autoimmune activity against an endocrine organ in association with at least one other endocrine or non-endocrine organ (13). The incidence of APS-4 is about 9 per 100,000 people. The syndrome usually manifests in early adulthood, with a mean age of onset of 23.1 years, although there is considerable variability, with some cases being diagnosed much later (11). Differential diagnosis is based primarily on component comorbidities that do not match any of the other APS/MAS (5, 6). The most common autoimmune conditions associated with APS-4 include diabetes mellitus type 1 and celiac disease, which are evenly distributed between the sexes. It is noteworthy that diabetes mellitus type 1 serves as the main indicator of APS-4 in 78% of cases and is often the second manifestation of the syndrome in 9% of patients (1, 11). The condition does not suggest a specific follow-up time, as 50% of patients develop APS-4 within the first ten years of onset. Additionally, 5% of women with APS-4 experience premature ovarian failure, highlighting the syndrome’s impact on reproductive health (1, 14). Diagnosis of APS-4 is often delayed, with an average delay of 11 years from initial clinical evaluation to diagnosis, highlighting the challenges of early detection and the need for increased clinical awareness (1, 15).

APS-1 (also known as autoimmune polyendocrinopathy, candidiasis and ectodermal dystrophy; APECED) was unlikely here: none of the core triad (chronic mucocutaneous candidiasis, hypoparathyroidism, or Addison’s disease) was present, and *autoimmune regulator* (*AIRE*) testing was negative; thus, APS-1 was excluded (6, 16, 17). APS-2 (Addison’s disease + autoimmune thyroid disease ± diabetes mellitus type 1) was excluded because neither Addison’s disease nor autoimmune thyroiditis was present (18). APS-3 requires autoimmune thyroid disease; because our patient lacked thyroid autoimmunity, APS-3 was not diagnosed (19–22). IPEX syndrome typically affects male infants and is linked to *forkhead box P3* (*FOXP3*) variants; phenotype, sex, age at onset, and genetics did not fit our case (7, 9, 23–25).

Immunosuppressive treatment and insulin therapy brought improvement, but the aggressive nature of autoimmunity and the need to intensify insulin therapy during steroid therapy significantly complicated the stabilization of the disease. The implementation of modern technology, such as a personal insulin pump, and extensive family education proved crucial to the successful management of the patient. Although the genetic testing performed did not reveal known pathogenic changes, the uniqueness of the clinical presentation suggests the possibility that previously undescribed mutations or polymorphisms may be involved in the pathogenesis of the disease or the potential occurrence of copy number variations (CNV) (26, 27). A timely medical response can not only improve the patient’s quality of life but also reduce the risk of further multi-organ complications, making this case a valuable contribution to the scientific literature.

In APS, a prolonged asymptomatic phase is frequently observed, during which the only detectable marker is the presence of circulating autoantibodies. Early identification of these antibodies enables timely diagnosis of autoimmune endocrine disorders and may help prevent tissue damage as well as the subsequent loss of secretory function. Gatta et al. reported that, in patients with APS-4, the diagnosis was, on average, delayed by 13.5 years. In patients diagnosed with an autoimmune disease, continuous surveillance is warranted to monitor for the potential development of additional autoimmune conditions. At present, there is no universally applicable screening protocol for all autoimmune disorders that can be routinely implemented in clinical practice. The authors propose an individualized flowchart, adapted to each outpatient specialty clinic (endocrinology, diabetology, gastroenterology, rheumatology, and clinical immunology), intending to simplify the follow-up of the complex spectrum of autoimmune associations in daily practice. In this framework, the emphasis shifts from a single ‘driver’ disease to the perspective of the different clinical specialties. Patients initially diagnosed with a gastrointestinal disorder developed APS considerably earlier than other groups, highlighting the need for more intensive monitoring in the early years, as 50% of such patients progressed to APS within three years of follow-up. Based on this observation, they propose an ‘outpatient timeline’. At disease onset, a comprehensive screening panel should be performed, including antibody testing for the most commonly associated autoimmune conditions. Subsequently, patients with gastroenterological involvement should undergo annual evaluations during the first three years, followed by functional organ testing every three years for an additional six years. The most frequent comorbidities to be assessed in this group include diabetes mellitus type 1, Hashimoto’s disease, and Graves’ disease. Endocrinology outpatients should be re-evaluated annually for the first two years and subsequently every two years for the following six years, with surveillance directed at diabetes mellitus type 1, celiac disease, rheumatoid arthritis, chronic atrophic gastritis, systemic lupus erythematosus, vitiligo, Hashimoto’s disease, and Addison’s disease (30). Screening for Addison’s disease should also be recommended when another autoimmune condition is present, as undiagnosed autoimmune diseases can lead to life-threatening situations such as adrenal crisis, ketoacidosis, hypo-, or hypermetabolic crises (1, 28). Diabetic patients should be assessed annually for the first two years and every two years thereafter for Hashimoto’s disease, celiac disease, Graves’ disease, rheumatoid arthritis, chronic atrophic gastritis, and vitiligo, with additional monitoring for inflammatory bowel disease. Rheumatology outpatients should undergo annual assessment for the first two years, followed by triennial evaluations for Hashimoto’s disease, diabetes mellitus type 1, Graves’ disease, and chronic atrophic gastritis. After the completion of this structured, specialty-specific follow-up, lifelong surveillance with a five-year screening interval is recommended for all patients. Particular attention should be directed to the risk of premature ovarian failure, a condition with profound consequences for women’s health. Anti-Müllerian hormone is regarded as a reliable biomarker for the diagnosis and risk stratification of premature ovarian failure in female patients with coexisting autoimmune diseases (30).

The proposed follow-up for this patient includes annual screening for autoimmune thyroid disease (thyroid-stimulating hormone [TSH], free thyroxine [fT4], anti-thyroid peroxidase [TPO] antibodies, anti-thyroglobulin [TG] antibodies, as well as anti-TSH receptor antibodies [TRAb]) and yearly clinical evaluation for Addison’s disease, with targeted hormonal and antibody testing in case of clinical suspicion. Strict adherence to a gluten-free diet will be maintained, with periodic celiac serology, nutritional assessment, and vitamin monitoring (D, B12, folate). Routine hematological surveillance with complete blood count, immunoglobulin levels, lymphocyte subpopulations, and complement components (C3, C4) will be performed. Annual assessment for autoimmune gastritis and pernicious anemia is planned, along with periodic dermatological evaluation for vitiligo and alopecia areata. Given the risk of premature ovarian failure, anti-Müllerian hormone measurement is recommended as a sensitive biomarker for early detection (30).

The patient’s autoimmune hepatitis with cirrhosis (F4) and portal hypertension necessitates structured hepatology follow-up, including ALT, aspartate transaminase (AST), bilirubin, albumin, international normalized ratio (INR), alkaline phosphatase (ALP), gamma-glutamyltransferase (GGT; every 1–3 months initially, then every 3–6 months), and IgG levels to assess disease activity. Maintenance steroid therapy will be continued. Abdominal Doppler ultrasound or elastography will be scheduled every 6–12 months, while alpha-fetoprotein (AFP) measurement and liver ultrasound every 6 months will be implemented for hepatocellular carcinoma surveillance. Endoscopic evaluation will be performed every 1–2 years to assess for esophageal varices. Supplementation of fat-soluble vitamins, osteoporosis and sarcopenia prevention, as well as consideration of liver transplantation in the event of hepatic decompensation, remain integral components of care (29).

Given the chronic steroid therapy, preventive bone health strategies include adequate vitamin D (800–2000 international units [IU]/day) and calcium supplementation, nutritional balance, and age-appropriate physical activity. Surveillance with dual-energy X-ray absorptiometry (DXA) every 1–2 years and monitoring of bone turnover markers (osteocalcin, procollagen 1 N-terminal propeptide [P1NP], C-terminal telopeptide [CTX]) are recommended, with bisphosphonate therapy considered in the event of progression to osteopenia or osteoporosis (31, 31).

How this case differs from previous reports of APS-4: (i) first reported such a combination of APS-4 components; (ii) very early age (6 years), relative to the predominantly adult-onset spectrum; (iii) synchronous presentation of four autoimmune entities rather than the typical stepwise accrual over years; and (iv) cirrhosis already present at diagnosis. The case highlights the challenges of the diagnosis-therapy process; therefore, the role of an interdisciplinary approach to it.

## Conclusions

The presence of one autoimmune disease in a patient increases the risk of developing other autoimmune disorders. Therefore, when a patient is diagnosed with one autoimmune disease, active screening for other conditions within this group should be conducted. It is important to remember that the clinical presentation of the patient may often result from the overlap of symptoms of different diseases. Accurate diagnosis and timely initiation of treatment can prevent severe complications and significantly enhance the patient’s quality of life. Undiagnosed and untreated autoimmune diseases can lead to life-threatening conditions, such as diabetic ketoacidosis.

## Data Availability

The original contributions presented in the study are included in the article/**Supplementary Material**. Further inquiries can be directed to the corresponding authors.
