# Supplementary material for: Case Report: Autoimmune polyglandular syndrome type 4 involving diabetes mellitus type 1, autoimmune hepatitis, immune thrombocytopenia, and celiac disease
Source: Front Endocrinol (Lausanne). 2025 Oct 15;16:1655483. doi: 10.3389/fendo.2025.1655483 (PMC12568398; doi:10.3389/fendo.2025.1655483)
Supplement: Supplementary file 1 [file DataSheet1.pdf]

Supplementary material

Supplementary table S1. Localization of various autoantigens and associated diseases. Modified from Bossowski et al., 2023 (S1) with permission from the Publisher (Wydawnictwo Naukowe PWN S.A., PZWL Wydawnictwo Lekarskie, Warsaw, Poland). The cited reference was published in Polish, while its excerpt was translated and modified by the authors.

Diseases	Autoantigens	Tissues/Cells
Addison's disease	21-OH, 17-OH, P450 scc	steroidogenesis enzymes
alopecia areata	tyrosine hydroxylase	hair bulbs
atrophic gastritis	H^+/K^+-ATPase	gastric parietal cells
autoimmune hepatitis	P450 2D6, P450 2C9, P450 1A2	liver
celiac disease	tTG, DGP, endomysium	small intestine
diabetes mellitus type 1	GAD_{65}, IA-2, pancreatic islet cells, insulin, ZnT8	pancreatic beta cells
Graves' disease	TSH receptor	thyrocytes
Hashimoto's disease	TPO/TG	enzyme/protein mainly in thyrocytes
hypogonadism	17-OH, P450 scc	Leydig cells/theca folliculi
hypoparathyroidism	CaSR	parathyroid chief cells
pernicious anemia	IF	gastric chief cell
vitiligo	tyrosinase	melanocytes

17-OH, 17α-hydroxylase; 21-OH, 21-hydroxylase; CaSR, calcium-sensitive receptor; DGP, deamidated gliadin peptide; GAD_{65}, glutamic acid decarboxylase 65-kDa isoform; IA-2, insulinoma-associated protein 2; IF, intrinsic factor; P450, cytochromes P450; scc, side-chain-cleavage; TG, thyroglobulin; TPO, thyroid peroxidase; TSH, thyroid-stimulating hormone; tTG, tissue transglutaminase; ZnT8, zinc transporter 8.

Supplementary table S2. The proposed diagnostic panel for suspected autoimmune polyglandular syndrome (APS). Prepared following Bossowski et al., 2023 (S1) with permission from the Publisher (Wydawnictwo Naukowe PWN S.A., PZWL Wydawnictwo Lekarskie, Warsaw, Poland). The cited reference was published in Polish, while its excerpt was translated, modified, and tabularized by the authors.

Baseline tests
serum sodium, potassium, calcium, magnesium, and phosphorus levels, complete blood count, vitamin B_{12}, urea, plasma renin activity, Hb_{A1C}
liver function parameters

TSH, fT4, fT3, ACTH, FSH, LH, PTH, testosterone, estradiol, glucose, fasting morning cortisol
**Dynamic tests**
adrenal stimulation test with synthetic corticotropin (if anti-adrenal antibodies are present)
**Autoantibodies**
ICA, GADA, IAA, against IA-2, against ZnT8
against TPO, against TG, TRAb
against P450 enzymes (especially 21-OH and 17-OH)
against H^+/K^+-ATPase in gastric parietal cells and IF
against tTG, against DGP, EMA
**Molecular analysis of the *AIRE* gene** (only for APS-1)
***HLA* typing and subtyping** (mainly for research purposes)

17-OH, 17α-hydroxylase; 21-OH, 21-hydroxylase; ACTH, adrenocorticotropic hormone; *AIRE*, *autoimmune regulator*; APS-1, autoimmune polyglandular syndrome type 1; DGP, deamidated gliadin peptide; EMA, anti-endomysial antibodies; FSH, follicle-stimulating hormone; fT3, free triiodothyronine; fT4, free thyroxine; GADA, anti-glutamic acid decarboxylase autoantibodies; Hb_{A1c}, glycated hemoglobin A1c fraction; HLA, human leukocyte antigen; IA-2, insulinoma-associated protein 2; IAA, anti-insulin autoantibodies; ICA, anti-islet cell antibodies; IF, intrinsic factor; LH, luteinizing hormone; P450, cytochromes P450; PTH, parathyroid hormone; scc, side-chain-cleavage; TG, thyroglobulin; TPO, thyroid peroxidase; TRAb, anti-TSH receptor antibodies; TSH, thyroid-stimulating hormone; tTG, tissue transglutaminase; ZnT8, zinc transporter 8.

Supplementary table S3. A panel of genes related to inborn errors of immunity was analyzed in the study.

ACP5	*CD3G*	*DNMT3B*	*IL17F*	*MYSM1*	*RASGRP1*	*TBX21*
ACTB	*CD40*	*DOCK2*	*IL17RA*	*NBAS*	*RELB*	*TCF3*
ADA	*CD40LG*	*DOCK8*	*IL17RC*	*NBN*	*RFX5*	*TCIRG1*
ADAM17	*CD46*	*ELANE*	*IL1RN*	*NCF1*	*RFXANK*	*TCN2*
ADAR1	*CD55*	*EPG5*	*IL21*	*NCF2*	*RFXAP*	*TERC*
AICDA	*CD59*	*ERCC6L2*	*IL21R*	*NCF4*	*RHOH*	*TERT*
AIRE	*CD70*	*EXTL3*	*IL2RA*	*NCSTN*	*RLTPR*	*TFRC*
AK2	*CD79A*	*FAAP24*	*IL2RG*	*NFAT5*	*RMRP*	*THBD*
AP1S3	*CD79B*	*FADD*	*IL36RN*	*NFKB1*	*RNASEH2A*	*TICAM1*
AP3B1	*CD81*	*FASLG*	*IL7R*	*NFKB2*	*RNASEH2B*	*TINF2*
AP3D1	*CD8A*	*FCGR3A*	*INO80*	*NHEJ1*	*RNASEH2C*	*TIRAP*
APOL1	*CDCA7*	*FCN3*	*IRAK1*	*NHP2*	*RNF168*	*TLR3*
ARPC1B	*CEBPE*	*FERMT3*	*IRAK4*	*NLRC4*	*RNU4ATAC*	*TMC6*
ATM	*CECR1*	*FLG*	*IRF2BP2*	*NLRP1*	*RORC*	*TMC8*

ATP6AP1	*CFB*	*FOXN1*	*IRF3*	*NLRP12*	*RPSA*	*TMEM173*
B2M	*CFD*	*FOXP3*	*IRF7*	*NLRP3*	*RTEL1*	*TNFAIP3*
BACH2	*CFH*	*FPR1*	*IRF8*	*NOD2*	*RUNX1*	*TNFRSF11A*
BCL10	*CFHR1*	*G6PC3*	*ISG15*	*NOP10*	*SAMD9*	*TNFRSF13B*
BCL11B	*CFHR2*	*G6PD*	*ITCH*	*NSMCE3*	*SAMD9L*	*TNFRSF13C*
BLM	*CFHR3*	*G6PT1*	*ITGB2*	*ORAI1*	*SAMHD1*	*TNFRSF1A*
BLNK	*CFHR4*	*GATA2*	*ITK*	*OSTM1*	*SBDS*	*TNFRSF4*
BTK	*CFHR5*	*GFI1*	*JAGN1*	*OTULIN*	*SEMA3E*	*TNFRSF6*
C1QA	*CFI*	*GINS1*	*JAK1*	*PARN*	*SERPING1*	*TNFSF11*
C1QB	*CFP*	*HAX1*	*JAK3*	*PEPD*	*SH2D1A*	*TNFSF12*
C1QC	*CFTR*	*HELLS*	*KDM6A*	*PGM3*	*SH3BP2*	*TPP1*
C1R	*CHD7*	*HMOX*	*KMT2D*	*PIK3CD*	*SLC29A3*	*TPP2*
C1S	*CIITA*	*HOIL1*	*LAMTOR2*	*PIK3R1*	*SLC35C1*	*TRAC*
C2	*CLCN7*	*HOIP1*	*LAT*	*PLCG2*	*SLC46A1*	*TRAF3*
C3	*CLPB*	*HYOU1*	*LCK*	*PLEKHM1*	*SMARCAL1*	*TRAF3IP2*
C4A	*COPA*	*ICOS*	*LIG1*	*PMS2*	*SMARCD2*	*TREX1*
C4B	*CORO1A*	*IFIH1*	*LIG4*	*PNP*	*SNX10*	*TRNT1*
C5	*CR2*	*IFNAR2*	*LPIN2*	*POLA1*	*SP110*	*TTC37*
C6	*CSF2RA*	*IFNG*	*LRBA*	*POLE*	*SPINK5*	*TTC7A*
C7	*CSF2RB*	*IFNGR1*	*LYST*	*POLE2*	*SRP54*	*TYK2*
C8A	*CSF3R*	*IFNGR2*	*MAGT1*	*PRF1*	*STAT1*	*UNC13D*
C8B	*CTC1*	*IGHM*	*MALT1*	*PRKCD*	*STAT2*	*UNC93B1*
C8G	*CTLA4*	*IGKC*	*MAP3K14*	*PRKDC*	*STAT3*	*UNG*
C9	*CTPS1*	*IGLL1*	*MASP2*	*PSEN*	*STAT5B*	*USB1*
CARD11	*CTSC*	*IKBA*	*MCM4*	*PSENEN*	*STIM1*	*USP18*
CARD14	*CXCR4*	*IKBKB*	*MEFV*	*PSMB8*	*STK4*	*VPS13B*
CARD9	*CYBA*	*IKBKG*	*MKL1*	*PSTPIP1*	*STN1*	*VPS45*
CASP10	*CYBB*	*IKZF1*	*MOGS*	*PTEN*	*STX11*	*WAS*
CASP8	*DCLRE1B*	*IL10*	*MS4A1*	*PTPRC*	*STXBP2*	*WDR1*
CCBE1	*DCLRE1C*	*IL10RA*	*MSH6*	*RAB27A*	*TAP1*	*WIPF1*
CD19	*DKC1*	*IL10RB*	*MSN*	*RAC2*	*TAP2*	*WRAP53*
CD247	*DNAJC21*	*IL12B*	*MTHFD1*	*RAG1*	*TAPBP*	*XIAP*
CD27	*DNASE1L3*	*IL12RB1*	*MVK*	*RAG2*	*TAZ*	*ZAP70*
CD3D	*DNASE2*	*IL12RB2*	*MYD88*	*RANBP2*	*TBK1*	*ZBTB24*
CD3E						

Supplementary references

S1. Bossowski A, Beń-Skowronek I, Sawicka B. '7. Zaburzenia wielogruczołowe. Autoimmunologiczne zespoły wielogruczołowe.', In: Pyrżak B, Walczak M, editors. Endokrynologia wieku rozwojowego. Warszawa: Wydawnictwo Lekarskie PZWL (2023). p. 523–546
